# Embedded IoT Design for Bioreactor Sensor Integration

**DOI:** 10.3390/s24206587

**Published:** 2024-10-12

**Authors:** Laurentiu Marius Baicu, Mihaela Andrei, George Adrian Ifrim, Lucian Traian Dimitrievici

**Affiliations:** 1 Electronics and Telecommunications Department, “Dunarea de Jos” University of Galati, 800008 Galati, Romania; mihaela.andrei@ugal.ro; 2 Automation and Electrical Engineering Department, “Dunarea de Jos” University of Galati, 800008 Galati, Romania; george.ifrim@ugal.ro; 3 Computer Science Department, “Dunarea de Jos” University of Galati, 800008 Galati, Romania; lucian.dimitrievici@ugal.ro

**Keywords:** bioreactor, embedded, ESP, IoT, Peltier module, turbidity

## Abstract

This paper proposes an embedded Internet of Things (IoT) system for bioreactor sensor integration, aimed at optimizing temperature and turbidity control during cell cultivation. Utilizing an ESP32 development board, the system makes advances on previous iterations by incorporating superior analog-to-digital conversion capabilities, dual-core processing, and integrated Wi-Fi and Bluetooth connectivity. The key components include a DS18B20 digital temperature sensor, a TS-300B turbidity sensor, and a Peltier module for temperature regulation. Through real-time monitoring and data transmission to cloud platforms, the system facilitates advanced process control and optimization. The experimental results on yeast cultures demonstrate the system’s effectiveness at maintaining optimal growth, highlighting its potential to enhance bioprocessing techniques. The proposed solution underscores the practical applications of the IoT in bioreactor environments, offering insights into the improved efficiency and reliability of culture cultivation processes.

## 1. Introduction

In science and industry, bioreactors are important tools in areas like pharmaceuticals, biotechnology, and environmental engineering. Bioreactors allow for the controlled growth of microorganisms, cells, or parts of cells under optimal conditions for extended periods. Among other factors, temperature control in bioreactors is very important for maintaining the high productivity and healthy growth of biological systems. Accurate temperature control can greatly affect metabolic rates, enzyme functions, and the overall health of cells [1,2].

The conventional ways of controlling temperature in bioreactors usually involve using external heating, like bioreactor heating mantles. For the cooling systems used in bioreactors and photo-bioreactors, complex meanders are designed, which can be expensive and require high maintenance. Also, these methods can be slow in adjusting the changes in temperature. The conventional systems often have a problem called thermal inertia, which causes delays in reaching the desired temperature and sometimes goes too far beyond it, which can harm the biological processes inside the bioreactor [3]. The emergence of thermoelectric technology, particularly Peltier modules, offers a promising solution for replacing the cooling or heating coils in bioreactors with Plexiglas walls, ensuring precise and rapid temperature control [4,5]. These modules, which utilize the Peltier effect, can both heat and cool by simply reversing the current direction, providing flexibility and efficient temperature regulation suitable for bioreactor environments [6].

The Internet of Things (IoT), for instance, represents the interconnection of a variety of objects or devices over the internet to communicate and interact with one another. These become “smart” devices by incorporating hardware, firmware, and software to help humans live more efficiently and interconnectedly. With public safety and privacy in mind, a critical reality is that this rapid technological progress has enabled transformative changes in our day-to-day lives by largely introducing convenience and automation [7], the most profound implication of which could arguably be an autonomous future.

The IoT framework encompasses a wide array of applications, from smart home systems that allow for the remote control of lighting, heating, and security, to advanced health monitoring systems that provide real-time data to both patients and healthcare providers [4,6]. In industrial environments, the IoT facilitates predictive maintenance and improves operational efficiency through real-time monitoring and data analytics [8].

With the continuous evolution of IoT technology, the traditional boundaries between different devices are diminishing, creating a cohesive and intelligent ecosystem. This shift not only enhances the quality of life by providing more personalized and responsive environments, but also offers substantial benefits in terms of energy management, security, and overall operational efficiency [9,10].

Utilizing the IoT, we are striving towards the advancement of a copresence with technology, which promotes innovations in many fields and enhances the interaction between people and their surroundings [11].

While the above developments promise great opportunities for many on their own, the IoT presents considerable challenges, such as the safety and privacy of its users, heterogeneity, and the explosion of data generated from the connected devices [10,12]. Such issues that are present in the market today must be resolved if the trends in the IoT consumption continue to rise [13,14].

An IoT network features a comprehensive structure, with diverse implementation possibilities that largely hinge on its overall topology. According to Miao et al. [15], the IoT architecture is typically divided into three distinct layers. The first is the physical layer, also known as the perception layer, which includes sensors responsible for detecting and collecting environmental data. This layer plays an important role in identifying the physical parameters of the surrounding smart objects. The second layer, known as the network layer, facilitates the connection between the smart devices within a network, enabling the transmission and processing of sensor data. The third and final layer is the application layer, which focuses on delivering application-specific services that interface between the users and smart objects. This layered architecture provides a clear framework for understanding how IoT systems operate and interact.

Furthermore, the incorporation of IoT systems into bioreactor systems allows for the real-time recording of various bioreactor control factors, such as temperature and turbidity. IoT systems make it possible to carry out management, data capture, and analysis from a distance and, thus, process management and optimization are improved [16,17].

Recent advancements in the IoT and sensor technologies have enabled the development of more integrated and automated bioreactor control systems. These systems not only improve the monitoring accuracy, but also allow for the better scalability and flexibility of various bioprocess applications [18,19]. The use of the IoT in bioreactor systems has been shown to enhance data accessibility and process transparency, leading to more informed decision making and improved bioprocess outcomes [20].

One parameter that can be measured in bioreactors is turbidity. Also described as the cloudiness of a fluid, it refers to the concentration of particles suspended in a fluid, including bacteria and cell debris, and is an important physical characteristic that should be monitored [21,22]. Turbidity is a critical parameter for various processes within a bioreactor. This is because it depends on biomass concentration control, which is vital for ensuring that the bioproduct consistency and quality are high [23].

In this paper, we build upon our previous work [24] on developing an IoT-based system for bioreactor temperature control by focusing on the practical application and effectiveness of this system in real-world scenarios. Currently, there is no developed system with the same structure as the one we propose. No commercially available bioreactor is controlled by an IoT microcontroller, capable of cooling or heating using the ESP module and bypass. These are essentially novel elements. Additionally, our system is a much cheaper option due to the low cost of the sensors used. This represents an advantage of the proposed solution. We investigate the growth rates of yeast cells under controlled temperature conditions using our system, which comprises an ESP32 microcontroller, a Peltier module, a turbidity sensor, and IoT capabilities for real-time data monitoring and control [25,26,27].

Through a series of experiments, we demonstrate how temperature regulation impacts the growth rates of these biological systems. The experimental results are analyzed to highlight the system’s efficiency, reliability, and potential advantages over traditional temperature control methods. Our research aims to provide valuable insights into the practical applications of advanced temperature control in bioreactors, contributing to enhanced bioprocessing techniques and outcomes.

## 2. Materials and Methods

The developed system is based on the ESP32 development board, which represents an updated version of the previously used ESP8266. The ESP32 is manufactured by the company Espressif Systems (Shanghai, China) and has a dual-core microprocessor with 32-bit LX6. The maximum frequency that it is capable of working at is 240 MHz. The board is suitable for IoT applications, and it has a built-in Wi-Fi module along with Bluetooth technology. The ESP32 development board features 38 pins, organized into various ports, enabling extensive connectivity. The board includes several General Purpose Input/Output (GPIO) pins, Pulse Width Modulation (PWM), analog-to-digital converters (ADC), and other interfaces like I2C, SPI, and UART. These interfaces facilitate the connection of multiple sensors and actuators, which can help develop needed applications. The ESP32 was chosen to be upgraded from the ESP8266 used in previous work due to its superior analog-to-digital conversion (ADC) capabilities, among other enhancements. The ESP8266 features a 10-bit ADC, providing a resolution of 1024 levels for converting analog signals to digital values. In contrast, the ESP32 has a 12-bit ADC, offering 4096 levels of resolution [28,29].

This higher resolution significantly enhances the precision of our turbidity measurements. With more bits, the ESP32 can detect finer variations in the sensor’s analog output, leading to more accurate and reliable turbidity readings. This improved accuracy is essential for optimizing the bioprocess, as it allows for more precise control over the concentration of suspended particles, including microorganisms and cellular debris. By choosing the ESP32, we ensure that our system delivers precise superior data quality, contributing to better monitoring and control of the bioreactor environment.

Accurate temperature control within the bioreactor is essential for maintaining optimal conditions for cell growth. To achieve this, we have used the DS18B20 digital temperature sensor, known for its high accuracy and reliability [30]. The DS18B20 can measure temperatures ranging from −55 °C to +125 °C with an accuracy of ±0.5 °C. It comes in more versions like stand-alone, board design circuit, and liquid-resistant, which is the version that we chose for the project. The sensor was connected to the ESP32 board via pin D4. To ensure stable and accurate readings, a 4.7 kohm pull-up resistor was used by connecting the signal pin and the power pin. Additionally, an LED was included in the circuit to provide a visual indication of the sensor’s operation. The DS18B20 communicates with the microcontroller using the 1-Wire protocol, which allows multiple sensors to be connected to the same data line, though in this article, only one sensor was used.

Monitoring the turbidity of the bioreactor culture provides insights into the concentration of suspended particles, such as microorganisms and cellular debris, essential for process optimization. We utilized the TS-300B turbidity sensor, which is designed to measure the cloudiness or haziness of a liquid. The TS-300B sensor was connected to pin D32 on the ESP32 board. This sensor works by emitting a light beam into the liquid and measuring the amount of light scattered by suspended particles. The sensor outputs an analog signal corresponding to the turbidity level, which the ESP32’s ADC can read. This real-time turbidity measurement enables precise monitoring and control of the bioprocess, allowing for adjustments to be made to maintain optimal conditions.

The TS-300B turbidity sensor typically has an accuracy error in the range of ±5% to ±10% of the measured value, depending on the calibration, quality of the sensor, and the specific conditions under which it is used. This translates to an error in NTU relative to the actual turbidity measurement. For instance, if the sensor measures 100 NTU, the potential error could range between ±5 NTU to ±10 NTU, resulting in a reading between 90 and 110 NTU.

For accurate turbidity measurements, we have used the TS-300B turbidity sensor that was calibrated in the process using solutions with known concentrations. The calibration process involved converting the sensor’s analog voltage output to turbidity units (NTU)

The calibration formula obtained from the regression analysis is

Turbidity (NTU) = −1604.28 × V + 3032.09 (1)

where V represents the voltage measured from the sensor.

The calibration formula was implemented in the microcontroller’s software to convert the analog voltage readings from the turbidity sensor to NTU values in real time. This process involved reading the analog value from the sensor, converting it to voltage, and then applying the calibration formula to determine the turbidity in NTU. To ensure that the NTU values remained within a reasonable range, the software included a check to set any negative NTU values to zero.

The final implementation enabled continuous and accurate monitoring of the bioreactor’s turbidity, facilitating control and optimization of the bioprocess. This comprehensive calibration and implementation ensured reliable turbidity measurements, enhancing the overall effectiveness of the bioreactor control system.

For temperature modulation within the bioreactor, a Peltier module was integrated into the system. Peltier modules are solid-state devices that can both heat and cool by reversing the current direction. They are compact, efficient, and capable of precise temperature control, making them ideal for bioreactor applications.

Given the Peltier module’s requirement for a high current supply of 10 amps, we have used a custom MOSFET control circuit. The MOS module board, initially designed for Arduino applications, was modified by replacing the default IRF520N MOSFET with an IRF3205 MOSFET. The IRF520N can handle a maximum continuous drain current of approximately 9.2 amps at 25 °C. While this is sufficient for many applications, it is not enough current for our Peltier module, which needs a current supply of 10 amps.

In contrast, the IRF3205 MOSFET is capable of handling significantly higher currents. It can support a maximum continuous drain current of up to 110 amps at 25 °C. This substantial increase in current capacity ensures that the Peltier module receives a stable and adequate power supply, even under high-demand conditions. In conclusion, IRF3205 is capable of handling higher currents, making it suitable for our application. The modified MOSFET control circuit was connected to pin D33 on the ESP32 board. This setup allows the microcontroller to regulate the current supplied to the Peltier module, enabling precise control of the bioreactor’s temperature. The Peltier module was mounted with radiators on both sides, with a fan on one side to dissipate heat or cold and a bypass on the other to facilitate the circulation of air or fluid through the bioreactor.

**Figure 1:**
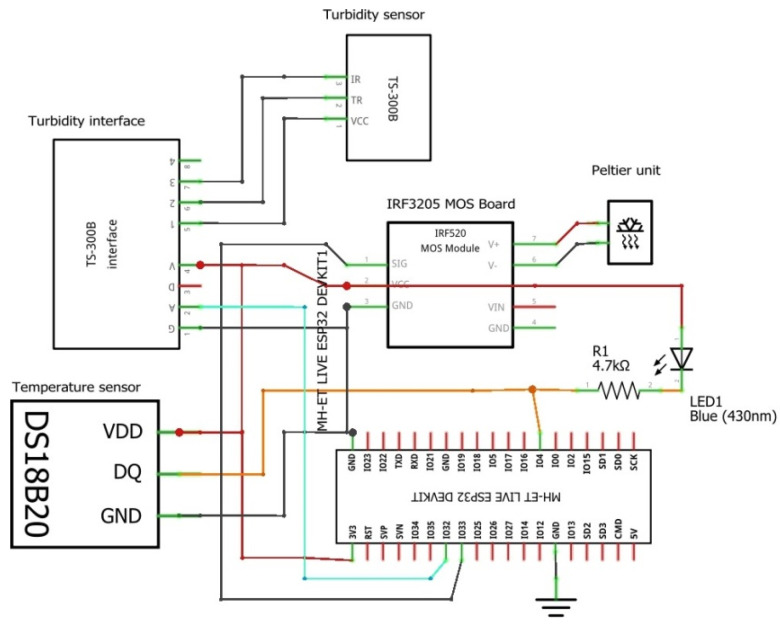
Electronic system design.

The overall schematic of the bioreactor control system integrates the ESP32 board, sensors, and Peltier module as in Figure 1. The DS18B20 temperature sensor is connected to pin D4, with an LED and 4.7-ohm resistor that lights up every time the ESP reads values from the sensor.

Figure 1 shows the complete wiring schematic of the system, detailing the connections between the ESP32 board, DS18B20 temperature sensor, TS-300B turbidity sensor, and the Peltier module.

**Figure 2:**
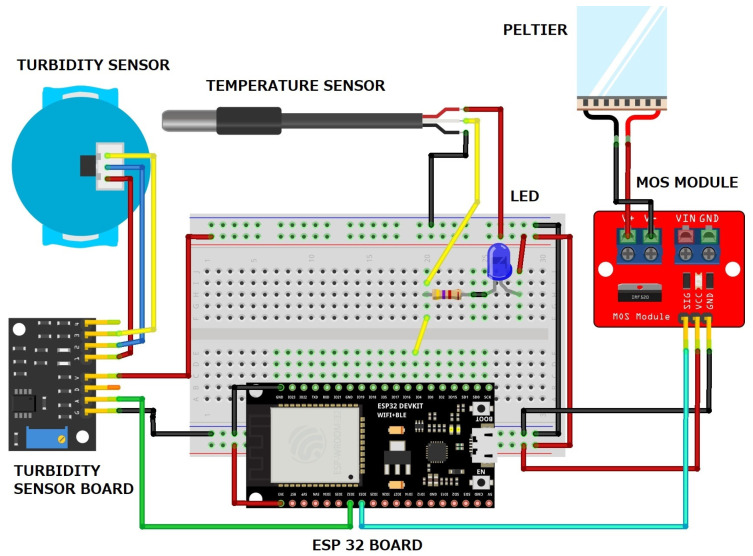
Breadboard schematic.

Figure 2 illustrates the breadboard setup, representing the physical layout of components and their connections, including the power control board and the Peltier module.

**Figure 3:**
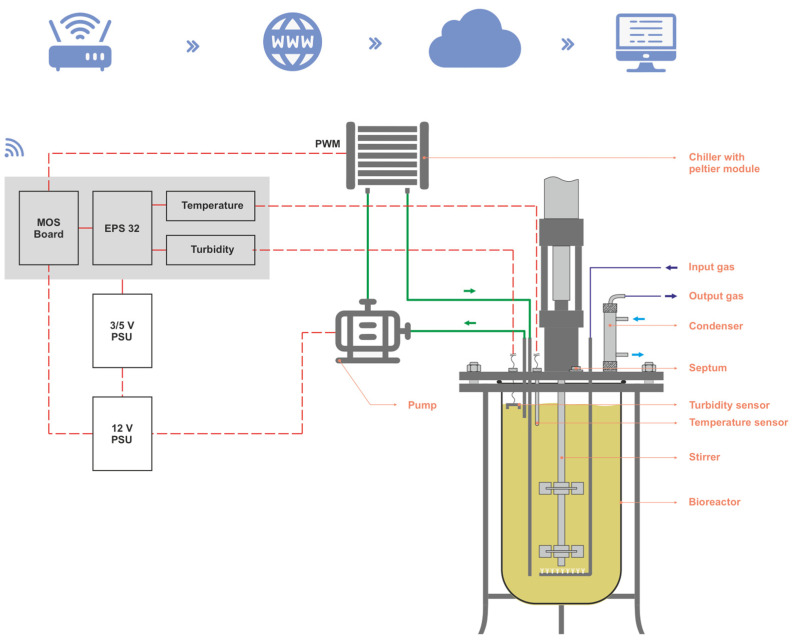
Bioreactor schematic.

Figure 3 illustrates our entire bioreactor system. The bioreactor vessel, the IoT microcontroller-based system, the chiller module with the Peltier and bypass, and the power supplies units are represented below. The Peltier module-based chiller maintains the bioreactor’s temperature, while the pump circulates fluids for cooling.

The TS-300B turbidity sensor interfaces with pin D32, allowing for continuous monitoring of culture turbidity. The Peltier module is controlled via a MOS module board, with the IRF3205 MOSFET connected to the microcontroller board, ensuring robust and reliable operation under high current conditions. The Vin and GND pins from the transistor board are inputs for the 12 V power source used to supply current to the Peltier module.

The developed system needs a power source that can deliver 12 V and a current of around 20 amperes so it can take the load of all the electronic components. Also, among the components one that can require the most current is the Peltier module.

This high-capacity power supply offers a stable and adequate power delivery, which is essential for good operation. The ESP32 board with its microcontroller is powered separately through its dedicated power supply board connected to the main 12 V system power supply.

This power supply board accepts a DC input ranging from 7 to 12 volts and provides selectable output voltages of either 3.3 or 5 V, depending on the requirements of the connected devices. In our setup, the power supply board is configured to output 5 V, which is then regulated to the necessary 3.3 V required by the ESP32.

**Figure 4:**
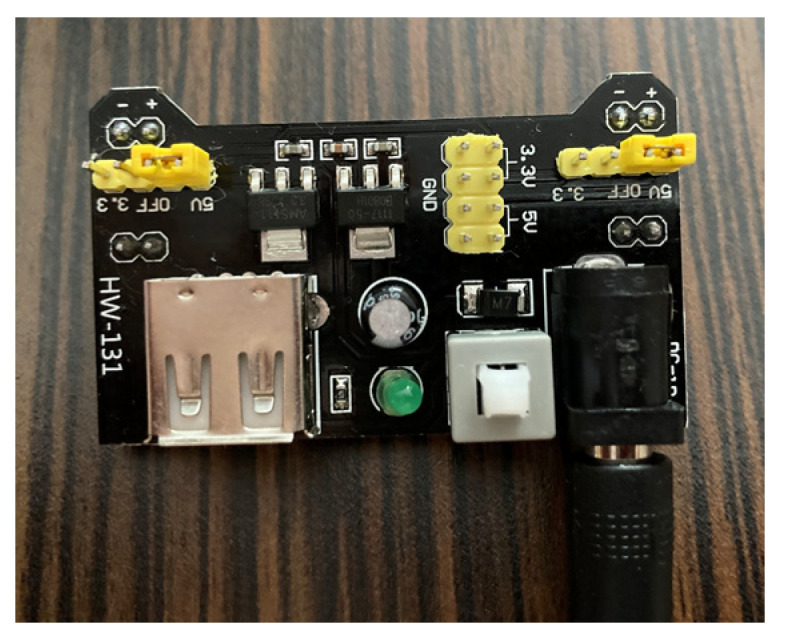
Power supply board.

This power supply board, despite its compact size, delivers a maximum current of 500 milliamperes, which is sufficient for the ESP32’s operational needs. The ESP32’s power requirements are relatively modest, and the 500 mA current capacity of the power supply board ensures stable operation without overheating or power interruptions. The choice of this power supply board was driven by its reliability and efficiency. It features a straightforward design, easy integration with the ESP32, and adequate power regulation capabilities, making it ideal for our bioreactor control system. Figure 4 below shows the power supply board used to power the ESP32.

The integration of IoT technologies has an important role in our system. It enables real-time data acquisition and remote monitoring. The ESP32 board’s Wi-Fi capability allows for seamless connectivity to cloud platforms, where data from the temperature and turbidity sensors is collected, analyzed, and visualized. This connectivity facilitates advanced process control and optimization, enhancing the overall efficiency and reliability of the bioreactor system.

For data transmission, the ESP32 board connects to a 2.4 GHz Wi-Fi network with IEEE 802.11 b/g/n standard [31], requiring the network’s SSID and password, which are programmed into the microcontroller’s firmware. The collected data can be sent to a cloud platform using HTTP or MQTT protocols, where it can be stored, analyzed, and displayed in real-time charts and graphs. This setup allows for remote monitoring and control, providing valuable insights into the bioprocess and enabling timely interventions if necessary. For our system, we have used Google Sheets as a platform for data collecting and storing.

In our experimental setup, yeast cells were cultivated within the bioreactor under controlled temperature conditions. The ESP32-based system continuously monitored and adjusted the temperature using the DS18B20 sensor and Peltier module. Simultaneously, the TS-300B turbidity sensor provided real-time data on cell growth and culture density. Data collected from these sensors were transmitted to Google Sheets for detailed analysis and process optimization. Through a series of experiments, the system’s performance was evaluated based on its ability to maintain optimal temperature conditions and monitor turbidity to identifying the culture state. The results demonstrate the system’s effectiveness, reliability, and potential advantages over traditional bioreactor control methods, contributing valuable insights into the practical applications of advanced IoT-based temperature control in bioprocessing.

## 3. Software Implementation

The program code for the ESP chip was made in Arduino IDE version 2.3.2. The bioreactor control system software is designed to manage hardware operations, process sensor data, and enable real-time monitoring through IoT capabilities. Developed with a focus on modularity and efficiency, the system handles key functionalities such as sensor data acquisition, data processing and calibration, control logic for temperature management, and IoT communication for data logging and monitoring.

**Figure 5:**
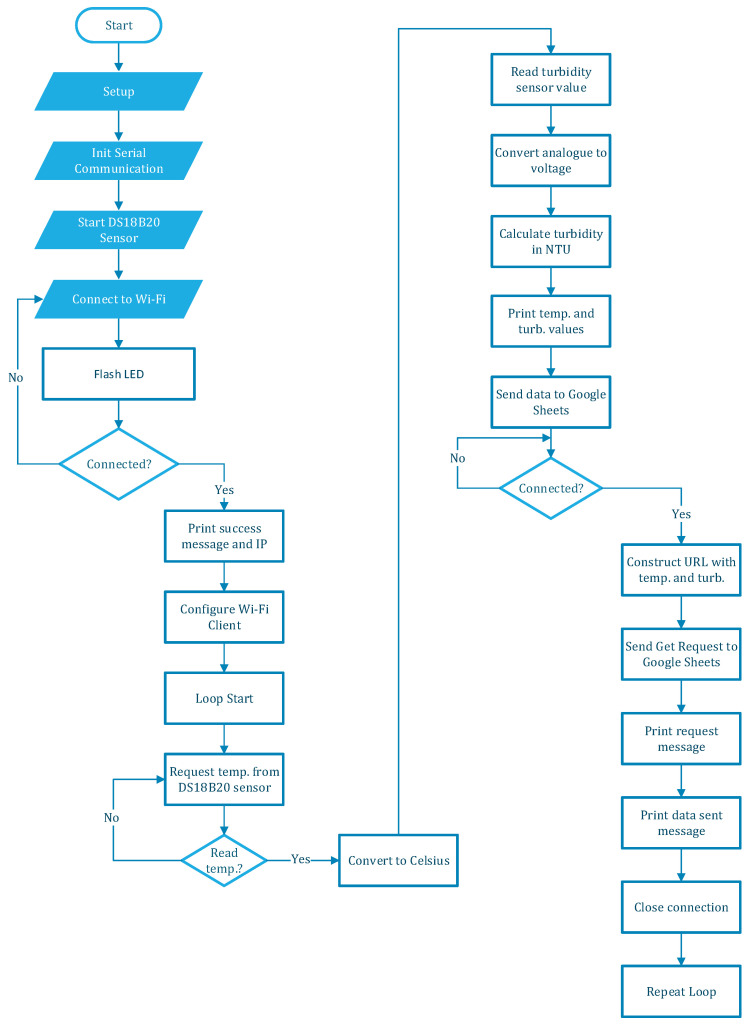
ESP32 main flowchart.

The software architecture includes several essential modules, beginning with the acquisition of sensor data. One module is responsible for reading temperature data from a DS18B20 sensor and turbidity data from a turbidity sensor connected to the microcontroller. Following data acquisition, the data processing and calibration module comes into play. This module processes the raw data from the sensors, applies calibration formulas, and converts the readings into meaningful units. Temperature readings are acquired, and analog turbidity values are converted to voltage and then to turbidity units (NTU). After connecting to the Wi-Fi and configuring the client, a loop for reading the temperature and the turbidity parameters is started and will collect data one by one at different time intervals. The next critical module is the control logic. This module manages the operation of a Peltier module to regulate temperature. Following Figure 5, the flowchart of the code developed for the ESP can be seen.

**Figure 6:**
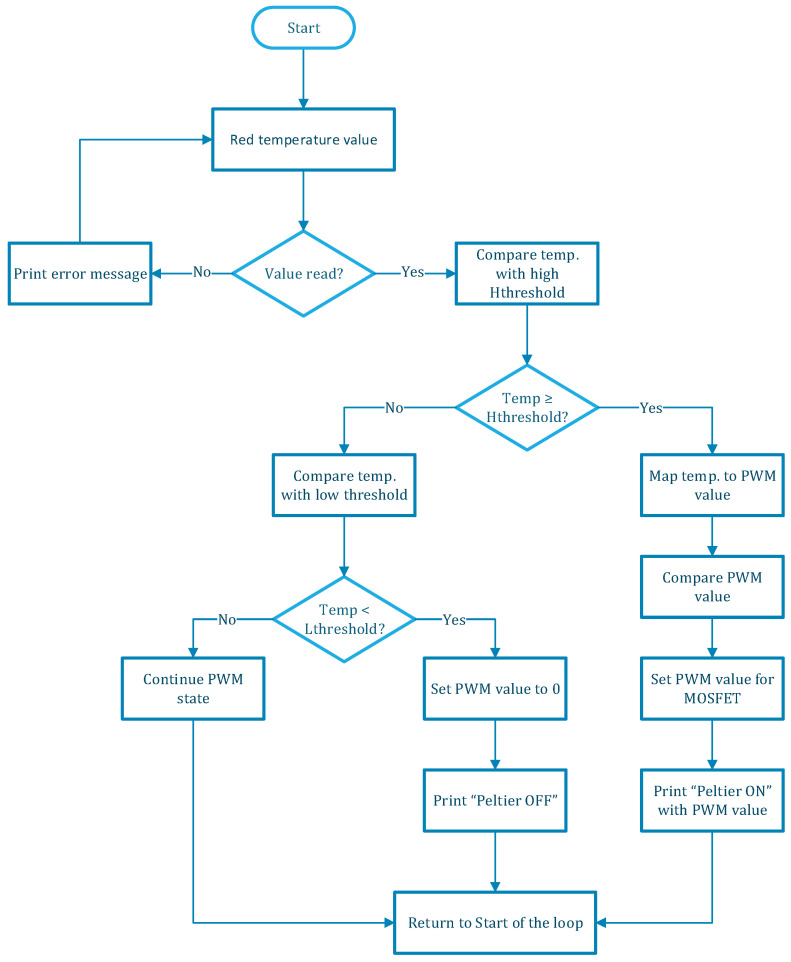
PWM flowchart.

By comparing the current temperature readings against predefined high and low thresholds, the system adjusts the Peltier’s activity to maintain optimal conditions within the bioreactor. The PWM control mechanism in our bioreactor control system is designed to manage the operation of the Peltier module, which is responsible for regulating the temperature. Figure 6 shows the flowchart of PWM control programmed in the ESP For our system, we have focused on cooling the bioreactor liquid.

This PWM value is then mapped based on the temperature, ensuring a proportional response. The calculated PWM value is then constrained to a maximum allowable limit to prevent overdriving the Peltier module.

Once determined, this PWM value is applied to the MOSFET controlling the Peltier module, effectively turning it on and adjusting its cooling intensity. The system also prints a message indicating that the Peltier module is on and displaying the current PWM value.

Alternatively, if the temperature is below the high threshold but above the low threshold, the system allows the current state to continue. However, if the temperature falls to or below the low threshold (set just below the high threshold to prevent frequent toggling), the PWM value is set to 0, turning off the Peltier module. This action is confirmed with a printed message indicating that the Peltier module is off.

By adjusting the PWM value based on real-time temperature readings, the control system ensures efficient and effective thermal regulation within the bioreactor. This approach not only maintains optimal temperature conditions but also enhances energy efficiency by activating the cooling mechanism only when necessary.

IoT communication is another important aspect of the system. It ensures that the sensor data that we collect is transmitted to a cloud platform, such as Google Sheets, for real-time monitoring and analysis. To facilitate this, the system establishes a secure HTTP connection to the Google Sheets script, constructs a URL with the sensor data as parameters, and sends an HTTP GET request to log the data. Throughout this process, status messages are printed to the serial monitor to confirm successful data transmission and connection status.

The implementation begins with initializing the necessary components. The serial communication is set up for debugging purposes, and the temperature sensor is initialized. The pin modes for the MOSFET controlling the Peltier module are configured, starting with the module turned off. The microcontroller then attempts to connect to a predefined Wi-Fi network using the provided SSID and password. During the connection process, the onboard LED flashes to indicate the status. Once the connection is established, the LED remains turned on, and the assigned IP address is printed to the serial monitor.

**Figure 7:**
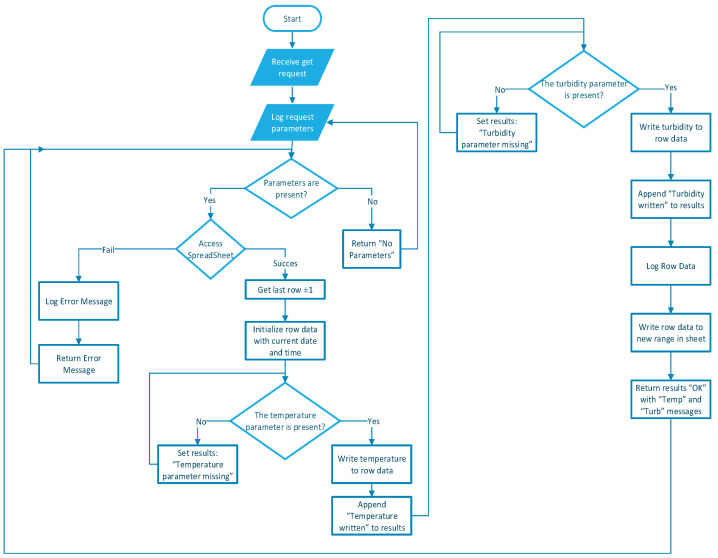
Google Sheets diagram.

In Figure 7, the flowchart of the code developed for Google Sheets can be analyzed.

With the sensors initialized and Wi-Fi connected, the system proceeds to read data from the temperature sensor and the turbidity sensor. The function *sensors.request temperatures* retrieves the latest temperature reading, which is then read and printed to the serial monitor.

Real-time monitoring and data logging are facilitated by sending the sensor data to a Google Sheets document using a secure HTTP connection. The *sendData* function constructs the URL for the Google Sheets script, including the temperature, turbidity, and PWM data as parameters. It then sends an HTTP GET request to log the data. During this process, the system continuously prints messages to the serial monitor, confirming each step and ensuring the data are accurately transmitted and recorded.

This approach ensures robust hardware management, accurate sensor data processing, effective temperature control, and reliable data logging for real-time monitoring and analysis of the bioreactor system. By leveraging IoT capabilities, the system provides a comprehensive solution for maintaining optimal conditions within the bioreactor, ensuring efficient and effective operation.

## 4. Results

This chapter presents the results obtained from our experimental evaluation of the IoT bioreactor system developed using a microcontroller. Our primary objective was to assess the impact of temperature regulation and turbidity monitoring on the growth rates of yeast cultures. The data collected throughout these experiments may offer valuable insights into the effectiveness of our system and its potential advantages over traditional bioreactor control methods.

To carry out the experiments, we configured a bioreactor system equipped with an ESP32 microcontroller, a DS18B20 temperature sensor, a TS-300B turbidity sensor, and a Peltier module for temperature control. The experimental setup was designed to monitor and regulate the environmental conditions within the bioreactor in real time. Data from the sensors were continuously recorded and transmitted to a Google Sheets document for analysis. Throughout the experimental period, we collected data at regular intervals, recording the temperature, turbidity, date/time, and control signal value. These measurements were needed to understand how temperature variations can influence the growth rate of yeast cultures and how turbidity reflects changes in biomass concentration. The experiments were divided into five distinct phases to comprehensively evaluate the temperature control capabilities of the system before introducing any biological cultures.

**Phase 1: Baseline Temperature Monitoring**

**Figure 8:**
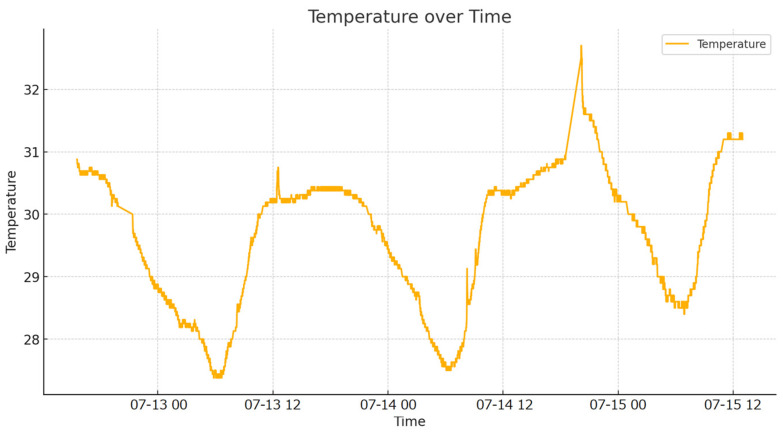
Temperature variations over three days.

Initially, the system was tested without growing any culture. This phase aimed to monitor temperature values over a period of three days to observe natural fluctuations and validate the cooling control mechanisms afterward. Data were collected every 30 s and sent to Google Sheets. The temperature variations between day and night were clearly visible in the graphical representation of the collected data in Figure 8. On the y-axis the temperature is displayed in degrees Celsius, and, on the x-axis, the time is displayed in dates and hours.

**Phase 2: Temperature Setpoint at 26 °C**

**Figure 9:**
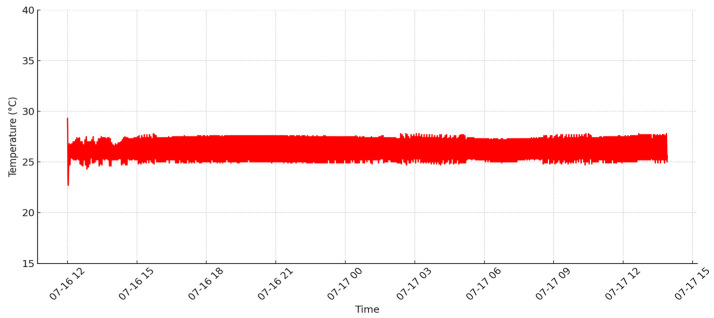
Temperature on day 4.

**Figure 10:**
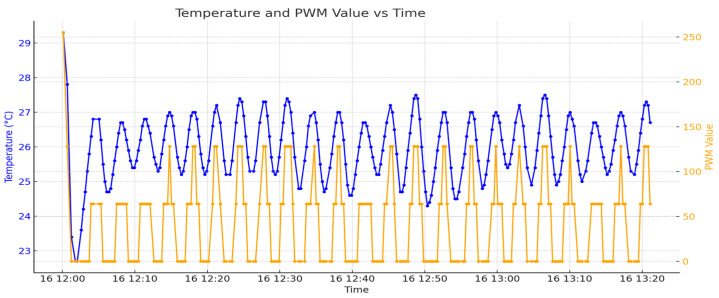
Temperature and PWM values on day 4.

On the fourth day, a temperature set point of 26 °C was established. The system’s ability to maintain this set point was evaluated with temperature data collected and analyzed. The Peltier unit was controlled using PWM signals to ensure the temperature at the desired set point. The temperature values can be seen in Figure 9 and the PWM control values in Figure 10.

**Phase 3: Temperature Setpoint at 23 °C**

On the fifth day, the temperature setpoint was adjusted to 23 °C. Similar to the previous phase, the system’s performance in maintaining this lower set point was monitored, with data collected and analyzed to confirm effective cooling and temperature stability. The set points were chosen to be appropriate for different culture cultivations in the bioreactor and to see how the system maintains these needed parameters [32,33,34].

**Figure 11:**
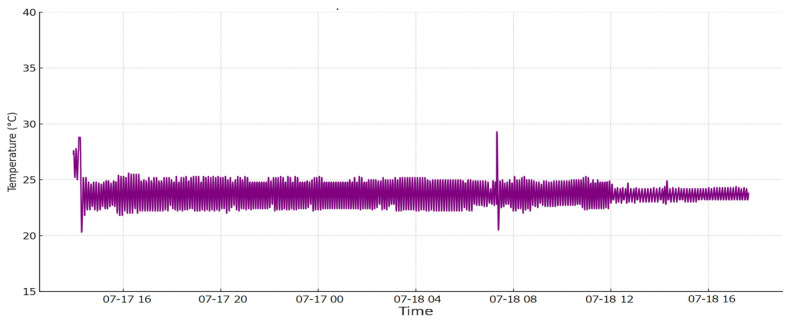
Temperature values plotted for day 5.

In Figure 11 the temperature values are plotted. Towards the end of the graph, we observe that temperature fluctuations have diminished, indicating improved precision.

This stabilization is attributed to a decrease in the environmental temperature where the bioreactor system was tested. Displayed values fluctuate on a second-by-second basis, but on average, the set point is maintained with minor variations that are negligible for the bioreactor culture and the overall process.

**Figure 12:**
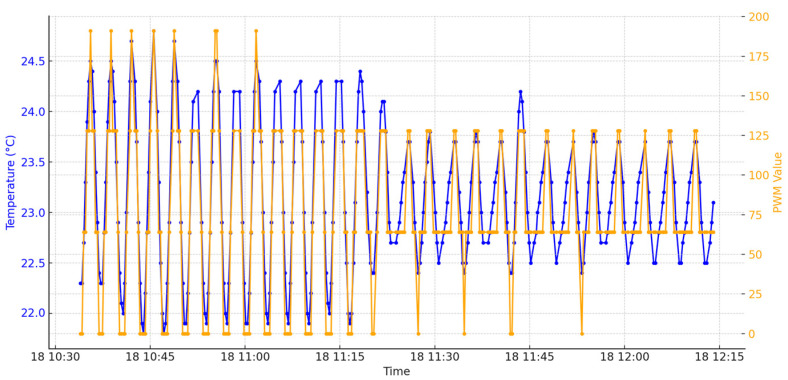
Temperature and PWM values on day 5.

Next, in Figure 12, temperature is represented by the blue color while the PWM values are represented by orange.

**Figure 13:**
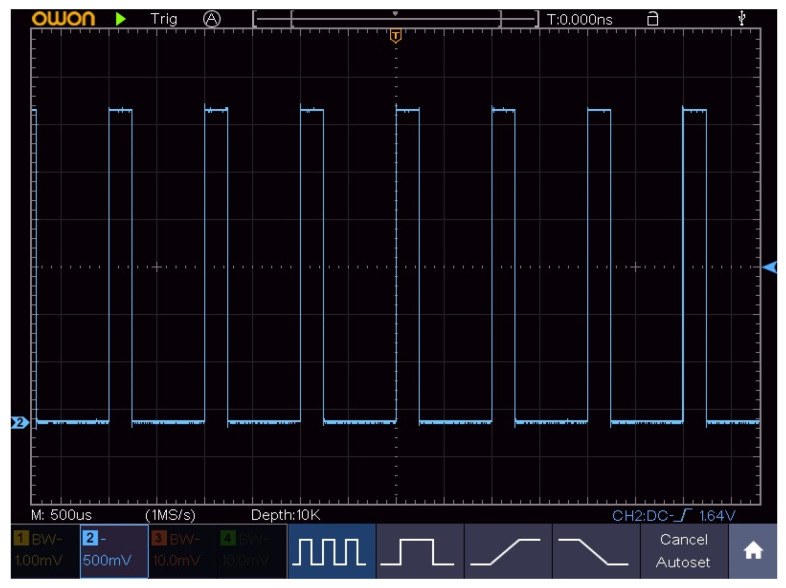
Oscilloscope image of PWM values.

There is a visible correlation between the temperature and PWM values. When the temperature increases or decreases, the PWM values adjust accordingly to maintain the desired temperature set point. This correlation demonstrates the effectiveness of the control system in responding to temperature changes by modulating the PWM signals. The graph may reflect the system’s adaptation to changing environmental conditions, such as a cooling trend. Reduced temperature fluctuations towards the end of the graph are due to a lower temperature in the environment. When this occurs the PWM will make adjustments in controlling and keeping the given temperature threshold by different mapping of the impulses. Figure 13 represents just a small enhanced portion of Figure 10 where the PWM signal is also plotted for better understanding.

The system’s performance appears to be adequate, as it successfully maintains the culture’s required conditions with minimal deviation, which is crucial for the bioreactor’s effectiveness and the biological process’s integrity. The duty cycle for the PWM signal was initially set to 255, which represents the maximum value and corresponds to a voltage output of 12 V. This configuration ensures that the Peltier module operates at its full capacity when required. However, through experimental analysis, we determined that the Peltier module requires a minimum threshold to initiate operation. Specifically, a minimum duty cycle of one-quarter is necessary to equate to a value of 64. This value corresponds to an approximate voltage of 3 V, which is sufficient to activate the Peltier module and start the cooling process.

Establishing this minimum duty cycle is critical for optimizing the energy efficiency and responsiveness of the bioreactor control system. By ensuring that the Peltier module only activates when the duty cycle reaches this calculated threshold, we can prevent unnecessary power consumption and improve the overall longevity of the system components. This threshold ensures that the cooling mechanism is engaged precisely when needed, maintaining the bioreactor environment within optimal temperature ranges without excessive energy use.

Figure 13 shows an image captured on the oscilloscope with approximately a 25% duty cycle on the microcontroller output pin for the input of the control MOS.

This fine-tuning of the PWM signal’s duty cycle is important to the system’s precision and adaptability. It highlights the importance of detailed calibration in achieving efficient and reliable bioprocessing. By carefully balancing the duty cycle parameters, we ensure that the Peltier module operates effectively, providing the necessary cooling while conserving energy.

**Power Consumption Analysis**

**Figure 14:**
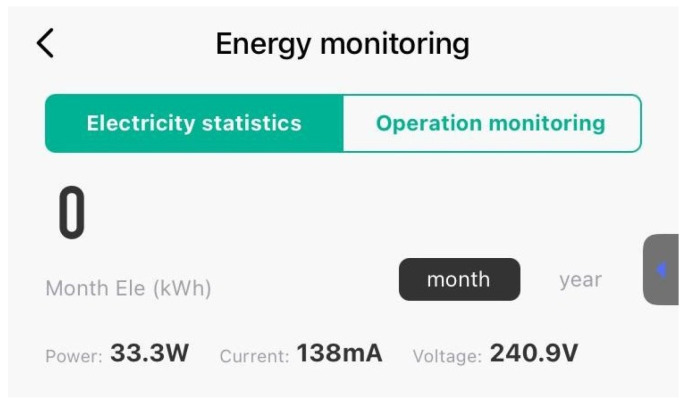
Power consumption at PWM 64.

In addition to temperature control, we monitored the system power consumption at different PWM values. This analysis helps to understand the efficiency and energy requirements of the Peltier unit under varying operational conditions. At a PWM value of around 64, the system power consumption was monitored as shown in Figure 14. This value represents a minimum cooling effort by the Peltier unit. The reading was made using IoT special measuring equipment which monitors the voltage from the power outlet in real-time, along with the current, and calculates the power consumed by the system. The equipment can also record the power consumption for a determined period but these features were not used because they are useful only for longer periods of time, with a minimum of one month.

In the image, we can observe an outlet voltage of about 240 V, a current of around 138 mA, and a computed power consumption of 33 watts of the entire system when the Peltier works at its minimum set state of the PWM.

**Figure 15:**
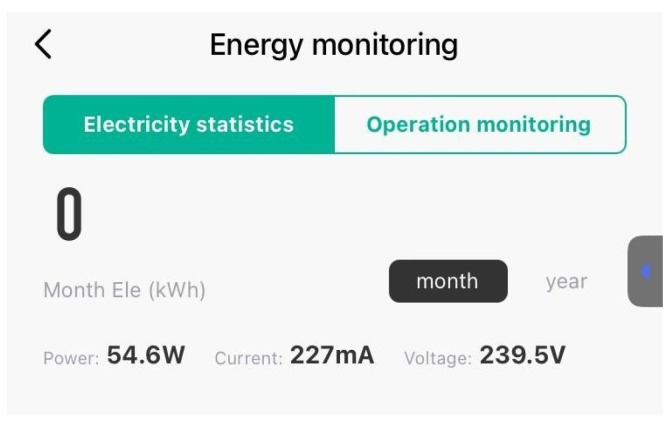
Power consumption at PWM 128.

At a PWM value of 128, which is a higher cooling effort, the power consumption was again monitored to evaluate the increase in energy usage. In Figure 15, it can be seen that the power outlet voltage has fluctuated slightly and it is now 239.5 V, while the current was raised to 227 mA and the computed power is around 55 watts.

**Figure 16:**
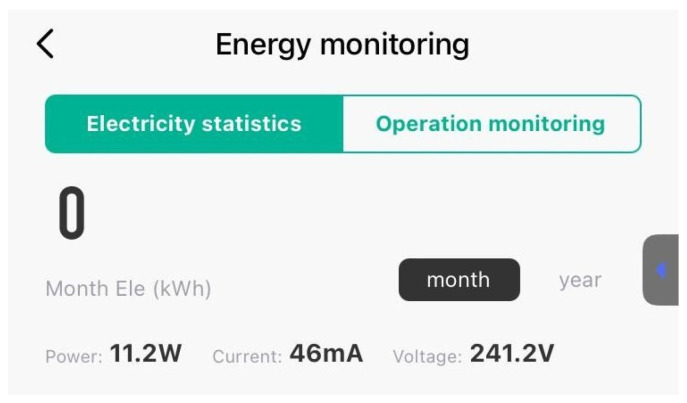
Power consumption at PWM 0.

The final measuring was at a PWM value of 0, when the Peltier unit was turned off, and the system’s baseline power consumption was recorded. This measurement helps in understanding the inherent power draw of the system without active cooling. In Figure 16, the outlet power measured is the highest around 241 V, with a drawn current of 46 mA and a computed power of 11 watts.

**Phase 4: Cell growth and turbidity measurement**

The next step was to introduce the system to a biological culture. For this step, we have chosen a culture of yeast cells from the *Saccharomyces cerevisiae* family. Yeasts from this family are frequently chosen for bioreactor testing and laboratory experiments due to their numerous advantages. Firstly, they are well-characterized model organisms with a fully sequenced genome, facilitating genetic and molecular studies. *Saccharomyces cerevisiae* also has an exceptional ability to ferment a variety of carbohydrates, efficiently converting them into ethanol and other useful byproducts, which is important for testing and optimizing fermentation conditions in bioreactors.

Moreover, *Saccharomyces cerevisiae* can be cultivated for biomass production, providing a significant yield of cellular material that is valuable for various biotechnological applications. These yeasts are robust and easy to cultivate under laboratory conditions, with well-defined environmental requirements and tolerance to a wide range of pH and temperatures. This flexibility allows for the precise replication and control of experiments, essential for obtaining reliable data.

*Saccharomyces cerevisiae* are cells that are considered safe for laboratory use and nonpathogenic. This yeast cells type reduces the risks associated with handling live organisms. These characteristics make *Saccharomyces cerevisiae* an ideal choice not only for testing bioreactor functionality, whether for fermentation or biomass production, but also for diverse biotechnological applications and fundamental research in cellular and molecular biology.

The experiment started with a young yeast culture placed in a bioreactor with approximately one liter of water. The necessary nutrients for growth were used, and the temperature was set at 29 degrees Celsius. Turbidity readings were taken at intervals of approximately 5 min, followed by averaging the readings.

**Figure 17:**
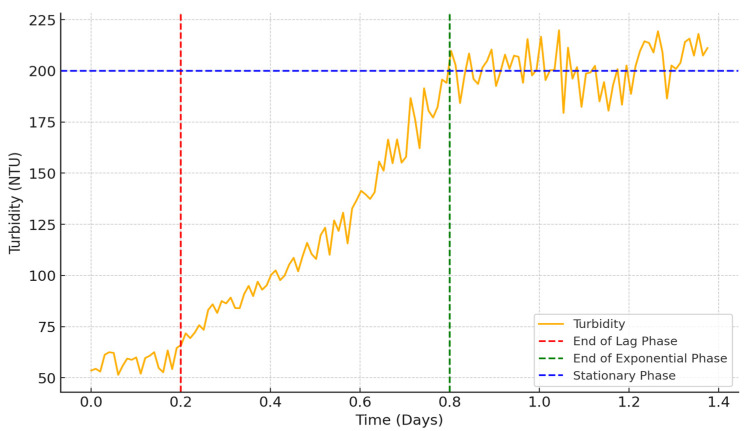
Turbidity of yeast cell culture.

The growth stages of the yeast cells are shown in Figure 17. It is known that yeast has several phases of growing. The first phase is called the Lag Phase, and, during this initial phase, yeast cells acclimate to their new environment. There is little to no cell division as the cells are metabolically active, synthesizing enzymes, proteins, and other molecules necessary for growth.

The ending of this phase can be observed in the image marked with a red line. In our testing, it lasted a few hours. Here, the turbidity remains constant or has a negligible increase.

The second phase is named the Log (Exponential) Phase and it is where the yeast cells begin to divide rapidly. The population size doubles at a constant rate, leading to an exponential increase in cell numbers. This phase continues as long as nutrients are abundant and waste products do not accumulate to inhibitory levels. The ending of this phase can also be seen in the graphic marked by a green line. Turbidity will rapidly increase here if the growing needs are met.

The third phase is called the Stationary Phase and describes the process as follows. The rate of cell division equals the rate of cell death, leading to a plateau in cell population size and a steady line in turbidity. The value for this phase was at around 200 NTU, which was measured with our turbidity sensor.

One last phase is the Death Phase in which nutrients become exhausted, and toxic waste products can accumulate, leading to an increase in cell death. The number of viable cells decreases over time. Usually, this phase is not encountered in the process of yeast culture growth in a bioreactor where biomass production is targeted unless performed deliberately. In our graph, this phase is not present. In this phase the turbidity gradually declines, although turbidity might not decrease proportionally because dead cells and cellular debris still contribute to it.

The results indicate that precise temperature control significantly affects the growth rate of yeast cultures. Maintaining the temperature within an optimal range (29–31 °C) resulted in good growth. The turbidity data enabled accurate estimation of biomass concentration, demonstrating the utility of real-time turbidity monitoring in optimizing bioprocess conditions.

**Phase 5: Decreasing temperature set point**

**Figure 18:**
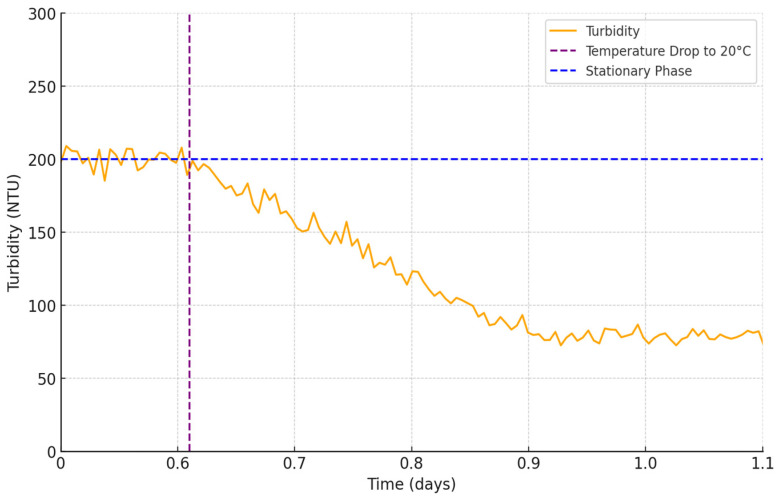
Turbidity at lower temperature.

In this phase of our research, we deliberately reduced the temperature of the bioreactor from 29 °C to 20 °C and kept the nutrient levels the same to investigate how this change would affect the growth rate of yeast cells. This new temperature set point was maintained consistently for approximately a day and a half and can be seen in Figure 18.

As we observed the system, it became evident that the turbidity of the culture began to decrease in response to the lower temperature. This reduction in turbidity can be attributed to the diminished activity and replication rate of the yeast cells at the cooler temperature. However, it is important to note that the turbidity did not drop to zero but rather stabilized at a certain level. This can be explained by the presence of yeast cell debris, few younger latent cells, and residual nutrients within the liquid medium, which continued to scatter light and contribute to the turbidity measurement.

Our experiments clearly demonstrated that the specific strain of yeast cells utilized in this study does not proliferate at 20 °C. Instead, we have observed different cell behavior; some of the older cells fail to the stress of the lower temperature, leading to cell death and decomposition, while other younger cells, entered a state of dormancy or latency. This phenomenon shows the adaptive strategies employed by yeast cells in response to unfavorable environmental conditions.

To provide a visual representation of these cellular changes, we captured a series of images at various stages of the experiment. The images of the yeast cells were captured, at different time intervals, outside the bioreactor chamber using the classical method with a microscope and a special digital camera attached to the microscope’s eyepiece.

**Figure 19:**
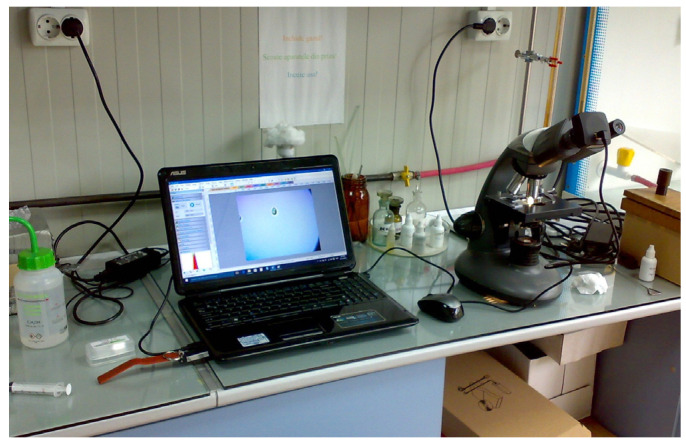
Microscope and camera setup.

**Figure 20:**
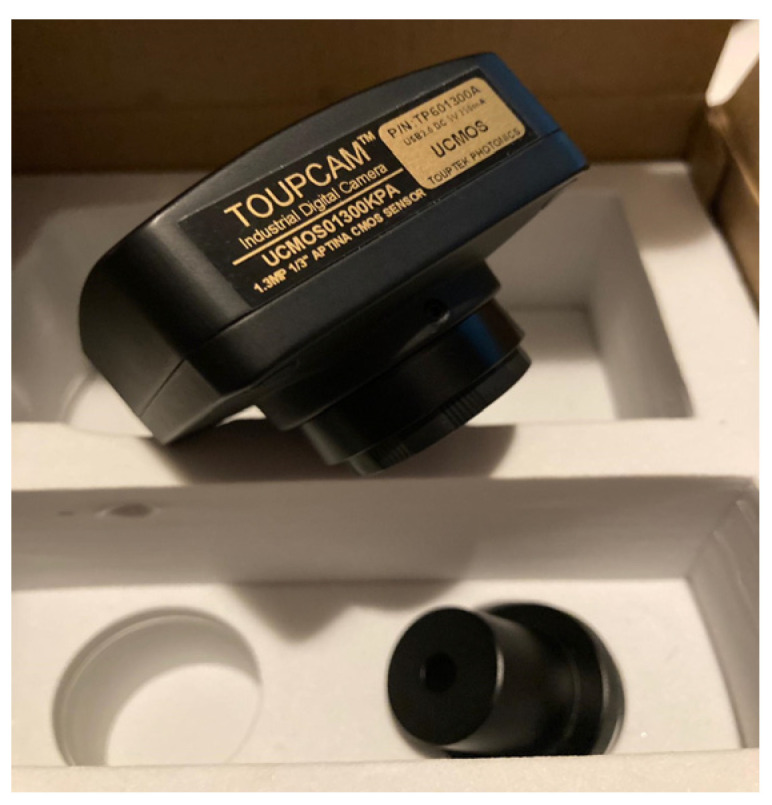
Microscope camera used.

The setup can be seen in Figure 19 and Figure 20. The microscope setup we have used has a 10× ocular lens and a 60× objective lens, having a total magnification of 600×. A microscope camera was used for image capturing, which was attached to the microscope’s ocular lens.

We have used the Toupcam Industrial Digital Camera TP601300A, which is designed for microscope use and has a 7.5 mm sensor and the same magnification as the microscope ocular of 10×. These images offer a detailed view of the cellular morphology and changes occurring within the yeast population over the course of the experiment. The following section presents these images, along with an analysis of the observed changes. Each image provides a snapshot of the cellular dynamics at different time points, illustrating the effects of the temperature reduction on yeast cell growth, viability, and overall health.

**Figure 21:**
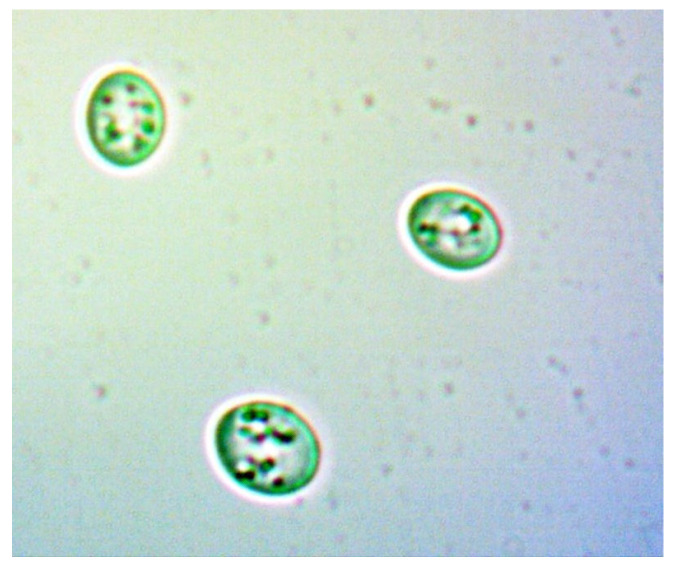
Young yeast cells sample 1.

**Figure 22:**
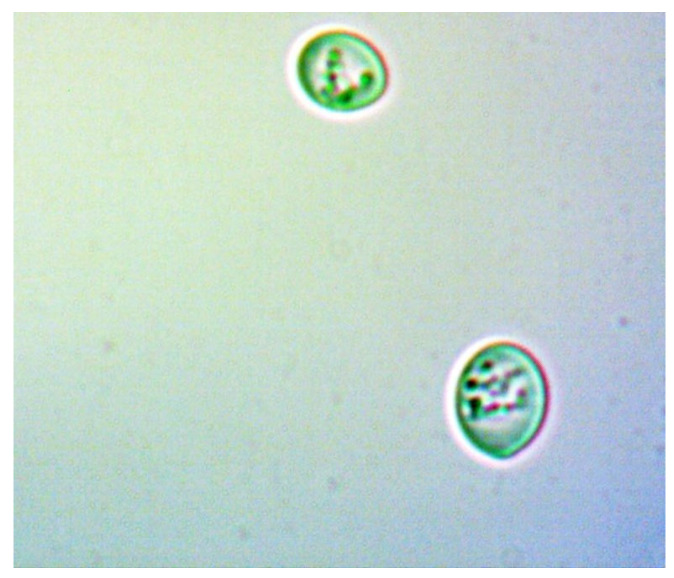
Young yeast cells sample 2.

**Figure 23:**
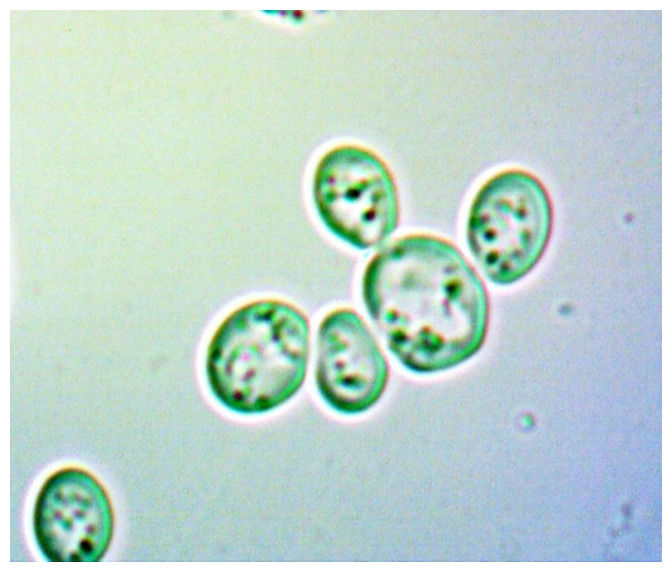
Multiplying cells sample 1.

**Figure 24:**
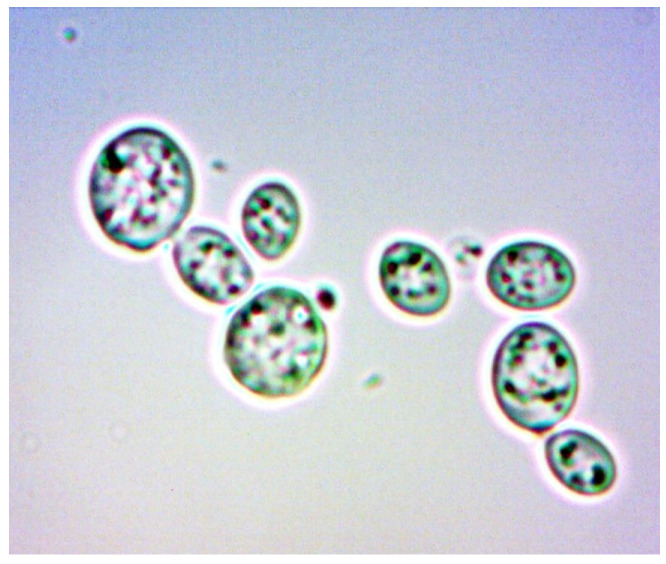
Multiplying cells sample 2.

**Figure 25:**
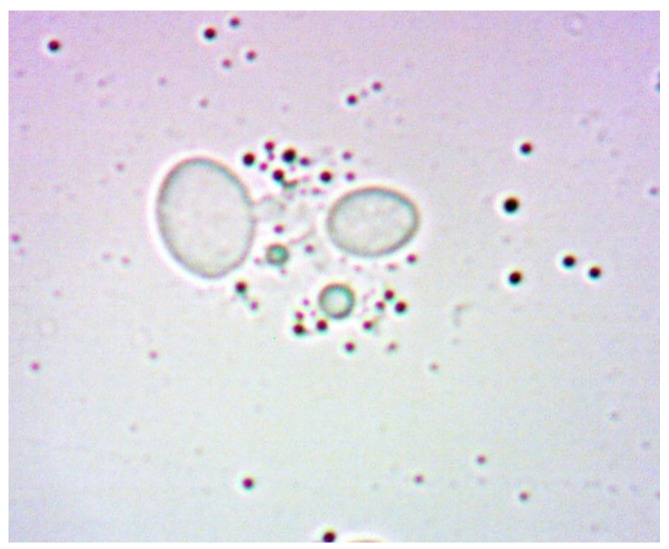
Decomposing cells sample 1.

**Figure 26:**
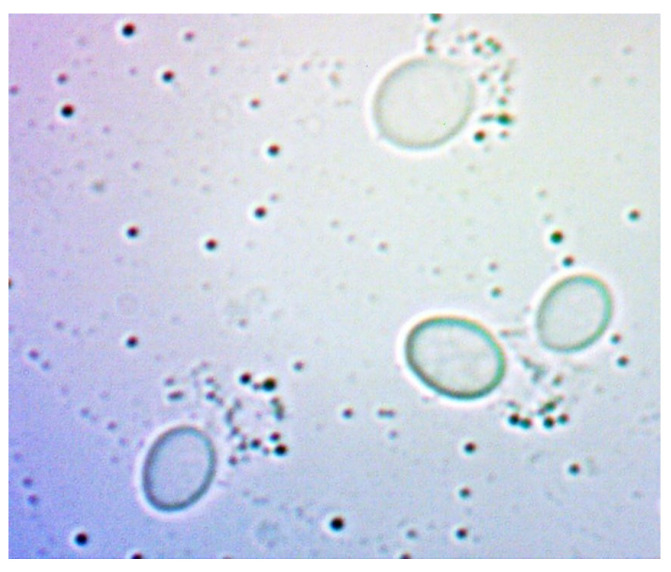
Decomposing cells sample 2.

Figure 21 and Figure 22 show young cells captured at the beginning of the experiments with sizes varying from 3 to 5 um. Continuing in Figure 23 and Figure 24, mature cells that are multiplying with sizes varying from 5 to 14 um for mature ones and even more for multiplying ones can be seen. In the next Figure 25 and Figure 26, we can see cells that are decomposing.

Also, considerable debris in the liquid can be seen. Due to the focus of the camera and different settings, to capture as clear as possible the cell and the membrane, mature and multiplying cells may look smaller or the same size as the young ones.

Our findings highlight the effectiveness of the ESP32-based control system in managing bioreactor conditions. However, there were some limitations, such as uncontrolled variations in other environmental factors and potential measurement errors. Future work could involve integrating additional sensors to monitor parameters like pH and dissolved oxygen and extending the experiments to other types of microorganisms.

By presenting these results, we aim to showcase the practical applications and benefits of advanced bioreactor control systems, contributing to more efficient and reliable bioprocessing techniques.

## 5. Conclusions

The proposed ESP32-based IoT system for bioreactor control demonstrated significant advancements in maintaining optimal environmental conditions for culture growth. The integration of precise temperature regulation using the DS18B20 sensor and the Peltier module, along with accurate turbidity monitoring via the TS-300B sensor, provided reliable real-time data for optimizing bioprocesses. The enhanced analog-to-digital conversion capabilities of the ESP32 enabled more precise measurements, contributing to better control over the bioreactor environment.

Our experimental results confirmed that the system effectively maintained set temperature points and accurately monitored turbidity, thereby supporting optimal yeast culture growth. The ability to transmit data to cloud platforms like Google Sheets for real-time monitoring and analysis further enhances the system’s utility. In conclusion, this paper highlights the practical benefits of integrating IoT technologies in bioreactor systems, demonstrating improved efficiency and reliability in culture cultivation. Future work should focus on extending the Peltier power so it can heat or cool more liquid, incorporating additional sensors to monitor other parameters and extending the application of this system to a broader range of microorganisms. The findings underscore the potential of advanced bioreactor control systems to contribute to more effective and sustainable bioprocessing methods.

## Data Availability

Some or all data, or codes that support the findings of this study, are available from the corresponding author upon reasonable request.
